# Lewy Body Dementia Research in Latin America: A Scoping Review

**DOI:** 10.1002/mdc3.70059

**Published:** 2025-04-21

**Authors:** Carlos Cano‐Gutiérrez, Salomón Salazar‐Londoño, Felipe Botero‐Rodriguez, Salomón Páez‐García, Salomón Giraldo, José Manuel Santacruz‐Escudero, Dag Aarsland, Miguel Germán Borda, COL‐DLB, Felipe Botero‐Rodriguez, Miguel Germán Borda, Omar Buritica, Catalina Cerquera‐Cleves, Maria Camila Gonzalez, Elkin Garcia‐Cifuentes, Alberto Jaramillo‐Jimenez, David Aguillon, Yamile Bocanegra, Beatriz Elena Munoz‐Ospina, Carlos Alberto Cano‐Gutierrez, Carlos Tobón, Hernando Santamaría‐García, José Manuel Santacruz‐Escudero, Dag Aarsland, Jorge Orozco, Salomon Salazar‐Londoño, Luis Carlos Venegas‐Sanabria, Salomón Páez‐García, Francisco Lopera, Juan Camilo Castro, Patrick Verhelst Forero, Alexandra Ferreiròs

**Affiliations:** ^1^ Intellectus Memory and Cognition Center Hospital Universitario San Ignacio Bogotá Colombia; ^2^ Semillero de Neurociencias y Envejecimiento, Ageing Institute, Medical School Pontificia Universidad Javeriana Bogotá Colombia; ^3^ SynaptIA – Inteligencia artificial para la investigación en salud mental Bogotá Colombia; ^4^ Centre for Age‐Related Medicine (SESAM) Stavanger University Hospital Stavanger Norway; ^5^ Departamento de Psiquiatría y Salud Mental Pontificia Universidad Javeriana Bogotá Colombia; ^6^ Centre for Healthy Brain Ageing, Institute of Psychiatry, Psychology, and Neuroscience King's College London London UK; ^7^ Department of Neurology Clínica Universidad de Navarra Pamplona Spain

**Keywords:** Lewy body disease, synucleinopathies, dementia, Latin America, review

## Abstract

**Background:**

Dementia research in Latin America (LA) has primarily focused on Alzheimer's Disease and Frontotemporal Dementia, while Lewy body dementia (LBD) has been largely forgotten.

**Objective:**

We aimed to review the available evidence on LBD in LA, offering a comprehensive perspective for understanding the lack of reports and the unique challenges and characteristics of this region.

**Methods:**

We carried out a scoping review in databases: PubMed, EMBASE, LILACS, and Web of Science. Original studies that included participants with LBD were analyzed.

**Results:**

Of the 1388 studies identified, 70 met the inclusion criteria for this review. Among them, 63 were cross‐sectional studies, three were cohort studies, two followed a case–control methodology, and only two were non‐randomized clinical trials. These studies primarily examined clinical manifestations, risk factors, neuropsychiatric and non‐motor symptoms, as well as cognitive impairment and its assessment in LBD within LA. Regarding geographical distribution, 52 studies were conducted in Brazil, seven in Argentina, the rest in Peru, Mexico, Colombia, Cuba, and Chile.

**Conclusions:**

LBD research in LA is underrepresented, with most studies being cross‐sectional, few utilizing a longitudinal design, and only two clinical trials, both of which lack rigorous methodology. Challenges include weak study designs, high heterogeneity, limited trials, and unclear differentiation within the LBD spectrum. Addressing these gaps requires increasing awareness, strengthening research capacity, securing funding, and fostering international collaboration.

Understanding dementia in underrepresented populations can improve the quality of life of those living with the disease and their care partners by reducing health and care inequities.^1^ In this sense, specifically for Latin America (LA), initiatives such as the Multi‐Partner Consortium to Expand Dementia Research in Latin America (ReDLat) have emerged targeting mainly Alzheimer's Disease (AD) and Frontotemporal Dementia (FTD).^2^ However, other etiologies, such as Lewy Body dementia (LBD), have remained largely neglected. Nevertheless, according to the position paper by D'Antonio et al,^3^ there are some recent initiatives in Brazil (The Brazilian Biobank for Aging Studies at University of São Paolo and The Federal University of São Paulo), Colombia (Colombian Consortium for the Study of Dementia with Lewy Bodies COL‐DLB), and Peru (the Peruvian Institute of Neurosciences) related to this disease.

LBD is the second most common cause of neurodegenerative cognitive disorders worldwide,^4^ accounting for 4.2% of all diagnosed dementia in primary care, and 7.5% in secondary care.^5^ People with LBD experience worse prognosis, lower quality of life, and higher mortality rates, compared to those with AD or other dementias. LBD represents a substantial burden for caregivers and the healthcare system.^6^ Moreover, disease‐modifying therapies are still not available, and few clinical trials are on course.^7^ However, recent major developments include the opportunity to accurately diagnose and classify the underlying pathology using seeding amplification assays, which has led to a new proposal for the biological definition of neuronal synuclein disorder, incorporating both Parkinson's disease dementia (PDD) and dementia with Lewy Bodies (DLB).^8^ This is expected to lead to renewed interest in these disorders including increased trial activity. Moreover, there is robust evidence demonstrating that the DLB diagnosis is frequently missed, and with every new consensus report, prevalence increases due to changes in sensitivity: 2005 criteria compared to 1996 criteria increased prevalence in the population and clinical case studies in more than 4%.^5^

Knowledge and research on LBD in LA are limited, likely due to a lack of available data, the complexity of the disease, underdiagnosis, and the resulting challenges in identifying and recruiting patients.^9^ Therefore, including underrepresented populations in research, and aiming for global harmonization represents a priority for LBD research. Specific consortiums oriented to this are ongoing, and a good example of this is the E‐DLB consortium.^10^ Also, since unique patterns of aging may exist in LA,^11^ there can be specific characteristics for LBD related to prevalence, clinical manifestations, or other aspects related to the disease.

We aimed to review the available evidence on LBD in LA, offering a view of the available reports and the unique challenges in the region. By examining study design, demographics, and methodology, we aim to identify key research gaps and support future studies to improve understanding and diagnosis of LBD.

## Methods

We carried out a scoping review according to the PRISMA Extension for Scoping Reviews (PRISMA‐ScR).^12^

### Selection Criteria

We included studies that explicitly inform the inclusion of people living with LBD in their analysis. The included studies were peer‐reviewed and consisted of cross‐sectional, longitudinal, or clinical trials. Studies published in English, Spanish, or Portuguese were considered.^13^

We excluded studies published uniquely in abstract format, mainly conference related, and multicenter studies with a sample from LA were excluded. Moreover, if patients with LBD were included in a broader category named “other dementias” or similar, that did not provide any information, were excluded. Case reports or series were also excluded.

### Search and Selection of Articles

PubMed platform, EMBASE (Elsevier), LILACS and Web of Science were the databases considered. We employed both controlled vocabulary and free‐text terms according to each database. The search strategy for these databases was related to LBD and LA as shown in Supplementary material 1.

The screening was performed with the Rayyan software, and independently conducted by two reviewers (SSL, SG), and a third reviewer was available for disagreements. We did not include any filters, and the last search was done in March 2024.

Studies’ findings were synthetized qualitatively with a descriptive approach, using a narrative description of the findings supported by a synthesis table.

## Results

**Figure 1:**
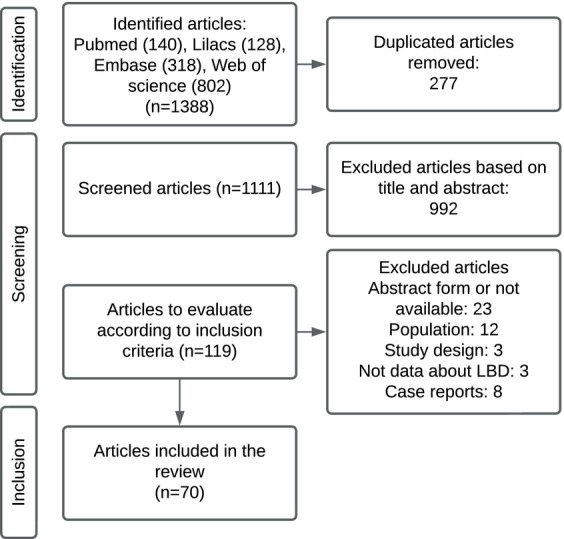
Prisma diagram.

A total of 1388 studies were identified, with 119 articles selected for full‐text evaluation. The primary reasons for exclusion were duplicate publications, lack of full‐text availability, and discrepancies in inclusion criteria. See Figure 1.

Overall, 2220 patients were included: 763 with LBD, 1220 with PDD, and 237 classified as LBD without further specification. The average number of participants per study was 28.3 (23.8 for DLB, 29.0 for PDD, and 47.4 for LBD). Sixty‐three papers had a cross‐sectional design,^14^, ^15^, ^16^, ^17^, ^18^, ^19^, ^20^, ^21^, ^22^, ^23^, ^24^, ^25^, ^26^, ^27^, ^28^, ^29^, ^30^, ^31^, ^32^, ^33^, ^34^, ^35^, ^36^, ^37^, ^38^, ^39^, ^40^, ^41^, ^42^, ^43^, ^44^, ^45^, ^46^, ^47^, ^48^, ^49^, ^50^, ^51^, ^52^, ^53^, ^54^, ^55^, ^56^, ^57^, ^58^, ^59^, ^60^, ^61^, ^62^, ^63^, ^64^, ^65^, ^66^, ^67^, ^68^, ^69^, ^70^, ^71^, ^72^, ^73^, ^74^, ^75^, ^76^ three were cohort studies,^77^, ^78^, ^79^ two had a case–control methodology,^80^, ^81^ and only two clinical trials were included.^82^, ^83^ Also, five papers included a neuropathological analysis in their methods.^36^, ^46^, ^47^, ^49^, ^68^

Regarding diagnostic criteria, among the articles including individuals with PDD (*n* = 44), 25 used the Movement Disorder Society (MDS) criteria, 12 applied the Diagnostic and Statistical Manual of Mental Disorders (DSM), four relied on a cognitive test, one used the Clinical Dementia Rating (CDR), and one did not specify the criteria used.

In terms of neuropathology, one study reported using a combination of Braak stages and CDR. Among the articles including patients with LBD (*n* = 36), nine applied the 3rd report of the Lewy Body Consortium, five used the 4th report, and six referenced the 1st report. Additionally, four studies used the DSM, one applied the Operational Criteria for Senile Dementia of the Lewy Body Type, and seven did not specify the diagnostic criteria used. For neuropathological studies, four studies reported using a combination of Braak stages and CDR.

**Figure 2:**
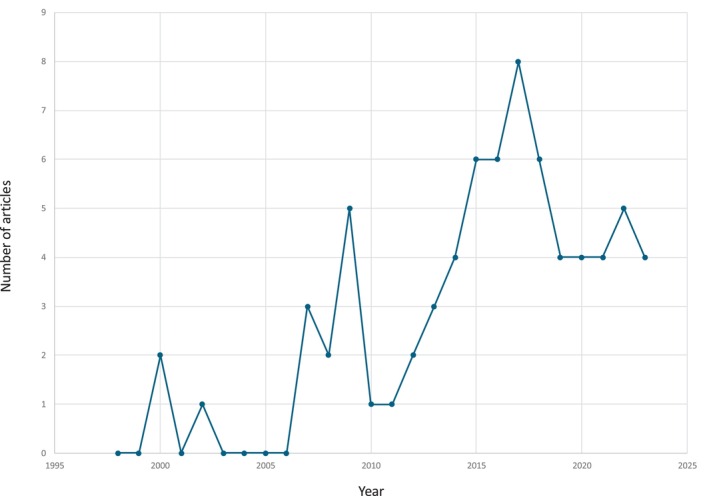
Number of published articles per year.

**Figure 3:**
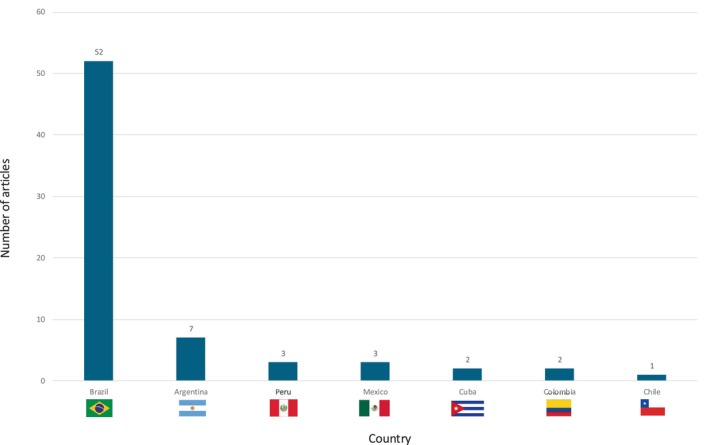
Number of published articles per country.

According to the publications over time, the data indicates a fluctuating trend in the number of papers, with an overall increase observed from the early 2000s to the mid‐2010s. The peak in publications occurred in 2017, after which there was a slight decline, but the number of publications has remained relatively stable in recent years (Fig. 2). Brazil is the leading country with 52 articles, followed by Argentina with seven articles, Peru and Mexico with three articles each, Colombia and Cuba had two articles each, and Chile with one article. This distribution highlights Brazil's dominant role in LBD research within the region, while other countries have a more limited output (Fig. 3).

Out of the studies reviewed, 36 articles had a focus on patients with LBD (1112 patients, with 470 LBD and 642 PDD). The mean sample size per study was 30.9, for DLB it was 36.2, and for PDD 24.7. These studies addressed various aspects of the condition, including risk factors, neuropsychiatric symptoms (NPS), non‐motor symptoms, cognitive impairment screening tools, biomarkers, and treatment (Table 1). The information related to articles that consider Lewy body disease as a subsample is in the supplementary material 2.

**TABLE 1:** Articles that were oriented to the study of patients with LBD

First author (year)	Country	Language	Study design	Number of patients with LBD	LBD diagnostic criteria	Category
de Oliveira, 2020^14^	Brazil	English	Cross‐sectional	DLB: 37 ‐ PDD: 14	MDS criteria and Fourth consensus of DLB consortium	Neuropsychiatric symptoms, non motor symptoms
Camargo, 2018^15^	Brazil	English	Cross‐sectional	PDD: 34	MDS criteria	Non motor symptoms
Garcia Basalo, 2017^16^	Argentina	English	Cross‐sectional	DLB: 75	Third consensus of DLB consortium	Cognitive impairment screening tools
Rocha, 2014^17^	Brazil	English	Cross‐sectional	PDD: 37	MDS criteria	Cognitive impairment screening tools
Golimstok, 2011^80^	Argentina	English	Case control	DLB: 109	First consensus of DLB consortium	Risk factors
Sobreira, 2015^18^	Brazil	English	Cross‐sectional	PDD: 17	MDS criteria	Cognitive impairment screening tools
Custodio, 2008^82^	Peru	Spanish	Open essay	PDD: 21 ‐ DLB: 12	DSM IV criteria, First consensus of DLB consortium	Treatment
Pérez, 2000^19^	Cuba	Spanish	Cross‐sectional	PDD: 19	DSM IV criteria	Risk factors
Machado, 2020^20^	Brazil	English	Cross‐sectional	PDD: 20 ‐ DLB: 22	MDS criteria	Neuropsychiatric symptoms
De Oliveira, 2023^76^	Brazil	English	Cross‐sectional	DLB: 27	Fourth consensus of DLB consortium	Biomarkers
Clavijo‐Moran, 2022^22^	Colombia	English	Cross‐sectional	NA	MoCA score < 18	Cognitive impairment screening tools
Sobreira, 2019^23^	Brazil	English	Cross‐sectional	PDD: 11	MDS criteria	Non motor symptoms
Sousa, 2023^24^	Brazil	English	Cross‐sectional	PDD: 22	MDS criteria	Cognitive impairment screening tools
Camargo, 2019^83^	Brazil	English	Non randomized clinical trial	PDD: 12	MDS criteria	Treatment
Camargo, 2016^26^	Brazil	English	Cross‐sectional	PDD: 39	MDS criteria	Neuropsychiatric symptoms
Reyes, 2009^26^	Argentina	English	Cross‐sectional	PDD: 13	MDS criteria	Cognitive impairment screening tools
Almeida, 2019^27^	Brazil	English	Cross‐sectional	PDD: 25	MDS criteria	Cognitive impairment screening tools
Camargo, 2018^28^	Brazil	English	Cross‐sectional	PDD: 33	MDS criteria	Cognitive impairment screening tools
Souza, 2016^29^	Brazil	English	Cross‐sectional	PDD: 40	MDS criteria	Risk factors
Tumas, 2016^30^	Brazil	English	Cross‐sectional	PDD: 29	MDS criteria + MOCA score < 21	Cognitive impairment screening tools
Schelp, 2016^31^	Brazil	English	Cross‐sectional	PDD: 46	MDRS	Risk factors
Campos, 2015^32^	Brazil	English	Cross‐sectional	PDD: 28	SCOPA‐COG Score ≤ 17	Risk factors
Custodio, 2013^33^	Peru	Spanish	Cross‐sectional	PDD: 23	DSM IV criteria	Risk factors
Schelp, 2012^34^	Brazil	English	Cross‐sectional	PDD: 19	MDRS	Risk factors
Tedrus, 2009^35^	Brazil	English	Cross‐sectional	PDD: 7	CDR	Risk factors
Gibson, 2023^36^	Brazil	English	Cross‐sectional	LBD: 60 ‐ AD+LBD: 28	Braak Parkinson's disease stage ≥3 and CDR	Neuropsychiatric symptoms
Oliveira, 2015^37^	Brazil	English	Cross‐sectional	PDD: 33	MDS criteria	Cognitive impairment screening tools
Calil, 2021^38^	Brazil	English	Cross‐sectional	DLB: 20	Fourth consensus of DLB consortium	Neuropsychiatric symptoms
de Oliveira, 2021^21^	Brazil	English	Cross‐sectional	DLB: 27	Fourth consensus of DLB consortium	Neuropsychiatric symptoms
Ferreira Camargo 2017^39^	Brazil	English	Cross‐sectional	PDD: 40	MDS criteria	Neuropsychiatric symptoms
Josviak, 2017^40^	Brazil	English	Cross‐sectional	DLB: 18	Third consensus of DLB consortium	Biomarkers
Oliveira, 2015^41^	Brazil	English	Cross‐sectional	DLB: 25 ‐ PDD: 14	Third consensus of DLB consortium and MDS criteria	Neuropsychiatric symptoms
Tabernero, 2017^42^	Argentina	Spanish	Cross‐sectional	PDD: 34	MDS criteria	Neuropsychiatric symptoms
Fonseca, 2013^43^	Brazil	English	Cross‐sectional	PDD: 12	MDS criteria	Biomarkers
Espínola Nadurille, 2007^44^	Mexico	Spanish	Cross‐sectional	DLB: 1	DSM IV criteria	Neuropsychiatric symptoms
Lourenco, 2021^45^	Brazil	English	Cross‐sectional	DLB: 9	Not specified	Biomarkers

Abbreviations: CDR, Clinical Dementia Rating; DLB, Dementia with Lewy Bodies; DSM, Diagnostic and Statistical Manual of Mental Disorders; LBD, Lewy Body dementia; MDRS, Mattis Dementia Rating Scale; MDS, Movement Disorder Society; MoCA, Montreal Cognitive Assessment; PDD, Parkinson's Disease Dementia; SCOPA‐COG, Scales for Outcomes in Parkinson's Disease Cognition.

### Risk Factors

Eight studies focused on risk factors. They documented history of adult attention deficit and hyperactivity disorder as a risk factor for LBD.^80^ In patients with Parkinson's disease (PD), the severity of the disease and the educational level of the patient were associated with PDD.^19^, ^32^ However, it is not clear if the initial cognitive profile can predict the cognitive prognosis of patients.^29^ Age appears to be related to cognitive decline in patients with PD,^34^ mainly affecting episodic memory, but in general, a large spectrum of neuropsychological performance was described.^31^, ^33^ Motor disability measured using the Hoehn and Yahr scale was associated with PDD.^35^

### Neuropsychiatric Symptoms

Ten studies focused on neuropsychiatric symptoms, and the Neuropsychiatric Inventory (NPI) was the most common tool. In patients with LBD from Brazil, based on a survival analysis, the duration of dementia was shorter specifically for severely impaired patients who had depression, based on the NPI, and were on antidepressants.^14^ In patients with DLB, neuropsychiatric symptoms appear to be more closely associated with impairments in visual organization than with linguistic features.^20^ It was also described that cases with dual pathology (DLB + AD) from Brazil had the highest risk of hallucinations, agitation, apathy, and total symptoms based on the NPI, and confirmed with histological post‐mortem studies,^36^ and patients with DLB may have a higher index of anosognosia compared to patients with AD,^38^ as well as a larger behavioral burden with hallucinations based on the NPI being inversely associated with Aβ42/Aβ38 and phospho‐Tau Thr181.^21^ Related to social cognition, the theory of mind is more impaired in patients with PDD compared to those with a behavioral variant of FTD.^42^ Moreover, hallucinations, apathy, dysphoria, anxiety, and aberrant motor behavior based on the NPI were the most significant to differentiate from AD.^41^

### Other Non‐motor Symptoms

Three studies examined non‐motor symptoms, specifically noting their higher prevalence in patients with DLB compared to those with PDD.^14^ For the last group, a correlation between female sex and olfactory changes has been described.^15^ Furthermore, the wake time after sleep onset and the number of state changes during sleep were associated with the global cognitive performance of patients with PDD.^23^

### Diagnosis: Biomarkers and Screening Tools

Five studies included data on biomarkers (4.2%). Three studies included data on cerebrospinal fluid (CSF) biomarkers,^21^, ^45^, ^76^ while another biomarker study had data on blood‐based ones.^40^ The last study included data related to electroencephalogram.^43^ Research indicated that CSF phospho‐tau Thr181 values in DLB were similar to those in AD, but not Aβ42, as patients with AD had lower values. Moreover, it was found that ratios performed better as diagnostic markers compared to cerebrospinal fluid amyloid‐β, tau, phospho‐tau Thr181, ubiquitin, α‐synuclein.^76^ Also, plasma butyrylcholinesterase activity (whose increased activity is associated with cognitive impairment) was evaluated as a possible biomarker for differential diagnosis between AD and LBD, finding a lower activity in the last.^40^ It was also reported that homovalinic acid and vascular endothelial growth factor were reduced in LBD patients.^45^ For electroencephalogram, delta and theta powers, beta frontal‐occipital inter‐hemispheric coherence, and alpha and beta frontal inter‐hemispheric coherence were highest in PDD patients.^43^ Also, we did not find any studies related to other biomarkers, such as dopamine transporter scan (DaTscan), myocardial scintigraphy or any magnetic resonance imaging analysis (structural or functional).

Eleven studies (9.2%) assessed cognitive impairment screening tools. For patients with LBD, the Argentine Lewy Body Association (ALBA) screening tool was described.^16^ Specifically for PDD, some tools were described: Addenbrooke's Cognitive Examination,^17^, ^18^, ^24^, ^26^ the Brazilian version of the Montreal Cognitive Assessment,^18^, ^27^, ^30^ Parkinson's Disease‐Cognitive Rating Scale (PD‐CRS),^22^ the Consortium to Establish a Registry for Alzheimer's Disease (CERAD) neuropsychological battery^28^ and the Movement Disorders Society checklist for the diagnosis of PDD.^37^ These studies focused on the validation, not on the population's cognitive performance.

### Intervention Trials

There were only two intervention trials, being no randomized placebo‐controlled trials. One non‐controlled open‐label study^82^ found beneficial effects in cognition, with an impact on activities of daily living, in patients with LBD, using cholinesterase inhibitors in 33 LBD patients after 6 months of follow‐up. Another non‐randomized trial compared the combination of cholinesterase inhibitors plus reality orientation therapy versus drug therapy alone in a 6‐month follow‐up. In 12 patients with PDD, it was found that combining therapy had better cognitive outcomes.^83^

### Lewy Body Dementia as a Subsample in Studies

There were some studies (*n* = 34, 48.6%) that included patients with LBD in a broader and more diverse sample,^46^, ^47^, ^48^, ^49^, ^50^, ^51^, ^52^, ^53^, ^54^, ^55^, ^56^, ^57^, ^58^, ^59^, ^60^, ^61^, ^62^, ^63^, ^64^, ^65^, ^66^, ^67^, ^68^, ^69^, ^70^, ^71^, ^72^, ^73^, ^74^, ^75^, ^77^, ^78^, ^79^, ^81^ with a total of 1107 patients (293 DLB, 578 PDD, and 236 LBD without discrimination). See Table 2. The mean sample sizes were 15.4 for DLB, 36.1 for PDD, and 47.4 for LBD. Patients were compared to AD in 14 studies, to FTD in eight studies, to VaD in 12 studies and to PD in 18 studies. Various aspects were addressed, including clinical manifestations like rapid eye movement (REM) sleep behavior disorder,^50^ cardiovascular symptoms,^62^ motor symptoms and response to levodopa challenge,^59^ and NPS.^58^ Additionally, researchers analyzed the neuropsychological profile, diagnostic tools, and biomarkers in LBD patients, including semantic verbal fluency,^48^ the relationship between electroencephalograms (EEG) and mild cognitive impairment (MCI) or dementia in PD patients,^63^ and the use of single‐photon emission computed tomography (SPECT) for differentiating synucleinopathies.^65^

**TABLE 2:** Articles that had patients with LBD in the sample

First author (year)	Country	Language	Study design	Number of patients with LBD	LBD diagnostic criteria	Category
Astolfi Neves, 2022^46^	Brazil	English	Cross‐sectional	DLB: 53	Braak Parkinson's disease stage ≥3 and CDR	Mortality
Suemoto, 2019^47^	Brazil	English	Cross‐sectional	LBD: *n* = 25 for <80 years and *n* = 37 for ≥80 years	Braak Parkinson's disease stage >3 and CDR	Neuropathology
Wajman, 2019^48^	Brazil	English	Cross‐sectional	DLB: 22	Fourth consensus of DLB consortium	Neuropsychological profile and diagnostic accuracy
Suemoto, 2017^49^	Brazil	English	Cross‐sectional	LBD: 87	Braak Parkinson's disease stage ≥3 // Third consensus of DLB consortium and MDS criteria	Neuropathology
Munhoz, 2014^50^	Brazil	English	Cross‐sectional	DLB: 50	Third consensus of DLB consortium	Clinical manifestations
Yamada, 2002^51^	Brazil	English	Cross‐sectional	PDD: 1	Third consensus of DLB consortium and DSM III‐R criteria	Prevalence estimation
Pineda, 2000^52^	Colombia	English	Cross‐sectional	LBD: 16	First consensus of DLB consortium	Prevalence estimation
Pessoa, 2022^53^	Peru	English	Cross‐sectional	DLB: 3 ‐ PDD: 2	DSM V Criteria	Non‐pharmacological treatment and follow up
Vale, 2018^54^	Brazil	English	Cross‐sectional	PDD: 5 ‐ DLB: 1	DSM IV Criteria	Prevalence estimation
de Moraes, 2017^77^	Brazil	English	Cohort	DLB: 27 ‐ PDD: 18	Not specified	Neuropsychological profile and diagnostic accuracy
Rodríguez‐Leyva, 2014^81^	Mexico	English	Case control	DLB: 12	Third consensus of DLB consortium	Biomarkers
Vieira, 2013^55^	Brazil	English	Cross‐sectional	DLB: 8	First consensus of DLB consortium	Prevalence estimation
Rodríguez‐Violante, 2012^56^	Mexico	Spanish	Cross‐sectional	DLB: 10	Third consensus of DLB consortium	Prevalence estimation
Bottino, 2008^57^	Brazil	English	Cross‐sectional	DLB: 1 ‐ PDD: 1	DSM IV criteria	Prevalence estimation
Stella, 2009^58^	Brazil	English	Cross‐sectional	PDD: 13	DSM IV criteria and MDS criteria	Clinical manifestations
Rossi, 2016^59^	Argentina	English	Cross‐sectional	DLB: 1	Not specified	Clinical manifestations
Studart Neto, 2017^60^	Brazil	English	Cross‐sectional	DLB: 1	Not specified	Prevalence estimation
Libre Rodríguez, 2009^61^	Cuba	English	Cross‐sectional	DLB: 5 ‐ PDD: 33	DSM IV criteria and Operational criteria for SDLT	Prevalence estimation
Idiaquez, 2007^62^	Chile	English	Cross‐sectional	PDD: 11	DSM IV criteria	Clinical manifestations
Fonseca, 2009^63^	Brazil	English	Cross‐sectional	PDD: 7	DSM IV criteria	Diagnostic tools
Souza, 2016^64^	Brazil	English	Cross‐sectional	PDD: 41	MDS criteria	Supporting clinical tests
Fabiani, 2022^65^	Brazil	English	Cross‐sectional	DLB: 16	Fourth consensus of DLB consortium	Diagnostic tools
Taragano, 2018^78^	Argentina	English	Cohort	DLB: 28	First consensus of DLB consortium	Risk factors and predictors
Starkstein, 2007^66^	Argentina	English	Cross‐sectional	PDD: 62	DSM IV criteria	Supporting clinical tests
Chagas, 2015^67^	Brazil	English	Cross‐sectional	PDD: 24	DSM IV criteria and CSI‐D	Risk factors and predictors
Nunes, 2022^68^	Brazil	English	Cross‐sectional	LBD: 68	Braak Parkinson's disease stage ≥3 and CDR	Neuropathology
Hartmann, 2014^69^	Brazil	English	Cross‐sectional	DLB: 2	First consensus of DLB consortium	Biomarkers
Fernandes, 2015^79^	Brazil	English	Cohort	PDD: 51	Minimental State Examination ≤23	Mortality
Vale, 2023^70^	Brazil	English	Cross‐sectional	PDD: 37	BCSB, FAQ scores and DSM IV criteria.	Clinical manifestations
Munhoz, 2010^74^	Brazil	English	Cross‐sectional	DLB: 42	Third consensus of DLB consortium and DSM IV criteria	Prevalence estimation
Balestrassi, 2021^73^	Brazil	English	Cross‐sectional	LBD: 4	Not specified	Prevalence estimation
Rodríguez‐de‐Paula, 2018^71^	Brazil	English	Cross‐sectional	PDD: 230	MOCA score < 21	Non‐pharmacological treatment and follow up
Ferretti, 2018^72^	Brazil	English	Cross‐sectional	DLB: 1	Not specified	Economic analysis
Moscovich, 2017^75^	Brazil	English	Cross‐sectional	DLB: 10 and PDD: 42	Third consensus of DLB consortium and MDS criteria	Mortality

Abbreviations: BCSB, Brief Cognitive Screening Battery; CDR, Clinical Dementia Rating; DLB, Dementia with Lewy Bodies; DSM, Diagnostic and Statistical Manual of Mental Disorders; FAQ, Functional Activities Questionnaire; LBD, Lewy Body dementia; MDS, Movement Disorder Society; PDD, Parkinson's Disease Dementia; SDLT, senile dementia Lewy body type.

Two studies^64^, ^66^ specifically assessed the performance of clinical tests in LBD patients. One study^64^ evaluated the interlocking finger test, while another^66^ examined the mentation, behavior, and mood section of the Unified Parkinson's Disease Rating Scale.

Furthermore, CSF analysis indicated that phospho‐tau levels were significantly higher in AD compared to other dementias, such as LBD.^69^ Finally, neuropsychiatric symptoms, particularly depression, were identified as potential predictors of conversion to dementia in both DLB and PD patients.^67^, ^78^ (See Table 2, Supplementary material 3 and 4).

## Conclusions

We conducted a scoping review of the literature focused on LBD in LA. To the best of our knowledge, this is the first review to address this topic. Within the limited literature related to LBD, most studies focused on risk factors, clinical manifestations, and diagnostic approaches. Study limitations included small sample sizes, cross‐sectional design, and very few studies employed diagnostic biomarkers. This work contributes to identifying gaps, highlighting the need and opportunities for LBD research in the region.

Our results highlight a significant gap in scientific production between LA and other countries. Notably, the United States (US) published more articles on LBD in a single year than LA has in its entire research history (140 publications from 1993 to March 2024 in LA vs. 1777 in the US in 2023 alone, based on a PubMed search). Moreover, research from high‐income countries is more frequently published in journals with higher impact factors, further emphasizing the disparity in scientific output and the dissemination of findings between LA and wealthier regions.^84^ This gap is also evident within LA itself, with Brazil producing significantly more research than other countries in the region. A similar pattern has been observed in other neurodegenerative diseases, such as FTD.^84^ Additionally, the limited economic resources allocated to scientific research in the region further hinder progress in this field.

Our findings also reveal a lack of participation in global research, as supported by the fact that only one study included LA population in a clinical trial,^7^ which also represents a lack of participation in global research. This gap not only affects research development but also limits the implementation of prevention and treatment strategies, ultimately challenging the global translatability of findings. For instance, only seven LA countries, Chile, Costa Rica, Cuba, Mexico, Puerto Rico, the Dominican Republic, and Uruguay, have an established national dementia plan.^85^

There is also a big gap between LBD and other dementias, where production in LBD is extremely low when compared with AD or FTD.^84^ There is scarce evidence on DLB in underrepresented populations, where the issue may lie not in the low number of people with the disease, but in the limited general knowledge about it, despite LBD being the second most prevalent cause of dementia worldwide.^4^ A clear example of this is the similar pattern reported in other regions. In 2014, a systematic review^86^ was conducted to summarize the epidemiology of neurodegenerative diseases in sub‐Saharan Africa. Out of 144 studies included, only one was related to patients with DLB.^86^ In this sense, D'Antonio et al^3^ reported that DLB cohorts in LA, Africa, and the Middle East do not reflect its global population or the true prevalence.

Improving clarity and accessibility of diagnostic criteria is essential, from medical education to continuous training for healthcare professionals in both primary and specialized care. Early suspicion and accurate detection of LBD are crucial for timely treatment and for recruiting participants in research studies.

A key step in advancing dementia research in Latin America is the development of regional initiatives like COL‐DLB^9^ and ReDLat.^2^ These efforts focus on generating local data to address research gaps, improve representation in global studies, and strengthen international collaboration. Drawing from our experience, a way forward includes establishing standardized protocols, expanding research networks, and securing sustainable funding. Similar approaches have been successful in other disease areas, and aligning dementia research with these models could enhance impact.

The prevalence of LBD in Latin America may be influenced by several factors, including confusion with more dementias such as AD or, more commonly, vascular pathology, both of which have variable clinical presentations and contribute to misdiagnosis.^44^ Genetic diversity also plays a role. LA populations have varying degrees of European ancestry, averaging 50–60%, with some countries exceeding 70–80%.^87^, ^88^, ^89^ While genetic similarities suggest comparable prevalence rates to European countries, disparities in healthcare access, awareness, and diagnostic practices likely contribute to variations. Further research is needed to clarify these influences and ensure accurate recognition of LBD across different populations.

This review has some limitations. Despite efforts to include all studies, there might be inherent biases in the selection process, such as those not indexed in the selected databases, albeit we included Embase, used a comprehensive search strategy, did not include any filter related to published year, and articles in English, Spanish or Portuguese were considered. Also, variability across included studies in terms of population characteristics, interventions, outcomes, and study designs limited the ability to pool data, and the quality of primary studies varied, which might impact the overall reliability of the findings. Also, as different studies may use data from the same cohort, it is important to acknowledge that the number of patients included may be lower. We did not specify or differentiate between MCI and dementia in the results due to insufficient data on cognitive decline severity and dementia grading. Besides, our results may not be universally applicable due to the differences in study populations, settings, or measuring tools, limiting generalizability. However, to the best of our knowledge, this is the first review of LBD research in this region. We believe our study can complement existing research consortia,^3^, ^9^, ^10^ such as COL‐DLB,^9^ which is currently recruiting patients in three cities to monitor clinical and paraclinical variables, including biomarkers. Furthermore, this study can pave the way for new research collaborations that will benefit LBD patients in LA. These collaborations are essential for overcoming the challenges of conducting research in the region, such as limited funding and scarce resources.

Finally, we call for action to increase awareness and promote original research in this region. Some ongoing initiatives are being conducted in collaboration with global and European networks, such as E‐DLB and the LBD Professional Interest Area.^3^, ^9^, ^10^ However, we emphasize the need for clinical trials and well‐designed longitudinal studies to inform public policies based on local data. Currently, diagnostic and treatment guidelines rely on data from other countries and consortia, highlighting the urgency for generating region‐specific evidence.^3^

## Author Roles

(1) Research project: A. Conception, B. Organization, C. Execution; (2) Methodological Analysis: A. Design, B. Execution, C. Review and Critique; (3) Manuscript Preparation: A. Writing of the first draft, B. Review and Critique, C. Review and approval.

C.C.G.: 1A, 1B, 2C, 3B, 3C.

S.S.L.: 1A, 1C, 2B, 3A, 3C.

F.B.R.: 1B, 2A, 2C, 3B, 3C.

S.P.G.: 1B, 1C, 2B, 3A, 3C.

S.G.: 1B, 1C, 2B, 3A, 3C.

J.M.S.E.: 2C, 3B, 3C.

D.A.: 1A, 2C, 3B, 3C.

M.G.B.: 1A, 1B, 2C, 3B, 3C.

## Disclosures

**Ethical Compliance Statement:** We carried out a scoping review according to the PRISMA Extension for Scoping Reviews (PRISMA‐ScR). Being a scoping review, it did not need IRB approval, nor informed patient consent was not necessary for this work. We confirm that we have read the Journal's position on issues involved in ethical publication and affirm that this work is consistent with those guidelines.

**Funding Sources and Conflict of Interest:** The study funding was provided by the Norwegian Health Association. Also, funded by the National Institute for Health Research (NIHR) Biomedical Research Centre at South London and Maudsley NHS Foundation Trust and King's College London. The authors declare that there are no conflicts of interest relevant to this work.

**Financial Disclosures for the Previous 12 Months:** The authors declare that there are no additional disclosures to report.

## Supporting information


**Supplementary material 1.** Search strategy.
**Supplementary material 2.** Lewy body disease as a subsample in the study.
**Supplementary material 3.** Full data on articles that were oriented to the study of patients with LBD.
**Supplementary material 4.** Full data on articles that had patients with LBD in the sample.

## Data Availability

The data that support the findings of this study are available from the corresponding author upon reasonable request.

## Appendix A

Members of COL‐DLB are: Felipe Botero‐Rodriguez, Miguel Germán Borda, Omar Buritica, Catalina Cerquera‐Cleves, Maria Camila Gonzalez, Elkin Garcia‐Cifuentes, Alberto Jaramillo‐Jimenez, David Aguillon, Yamile Bocanegra, Beatriz Elena Munoz‐Ospina, Carlos Alberto Cano‐Gutierrez, Carlos Tobón, Hernando Santamaría‐García, José Manuel Santacruz‐Escudero, Dag Aarsland, Jorge Orozco, Salomon Salazar‐Londoño, Luis Carlos Venegas‐Sanabria, Salomón Páez‐García, Francisco Lopera, Juan Camilo Castro, Patrick Verhelst Forero, Alexandra Ferreiròs.
