# Supplementary material for: Lewy Body Dementia Research in Latin America: A Scoping Review
Source: Mov Disord Clin Pract. 2025 Apr 21;12(8):1053–65. doi: 10.1002/mdc3.70059 (PMC12371463; doi:10.1002/mdc3.70059)
Supplement: Supplementary file 1 — Supplementary material 1. Search strategy. Supplementary material 2. Lewy body disease as a subsample in the study. Supplementary material 3. Full data on articles that were oriented to the study of patients with LBD. Supplementary material 4. Full data on articles that had patients with LBD in the sample. [file MDC3-12-1053-s001.docx]

**Supplementary material 1.** Search strategy

PubMed: (("dementia with lewy bodies"[All Fields]) OR ("lewy body disease"[All Fields]) OR ("parkinson s disease dementia"[All Fields])) AND (("latin america"[All Fields]) OR ("argentina"[All Fields]) OR ("bolivia"[All Fields]) OR ("brazil"[All Fields]) OR ("chile"[All Fields]) OR ("colombia"[All Fields]) OR ("costa rica"[All Fields]) OR ("cuba"[All Fields]) OR ("ecuador"[All Fields]) OR ("el salvador"[All Fields]) OR ("guatemala"[All Fields]) OR ("haiti"[All Fields]) OR ("honduras"[All Fields]) OR ("mexico"[All Fields]) OR ("nicaragua"[All Fields]) OR ("panama"[All Fields]) OR ("paraguay"[All Fields]) OR ("peru"[All Fields]) OR ("republica dominicana"[All Fields]) OR ("dominican republic"[All Fields]) OR ("uruguay"[All Fields]) OR ("venezuela"[All Fields]) OR ("puerto rico"[All Fields])).

LILACS: demencia por cuerpos de lewy OR enfermedad por cuerpos de lewy OR demencia por enfermedad de parkinson AND ( db:("LILACS"))

Web of science: (ALL=(Dementia with Lewy bodies) OR ALL=(Lewy body disease) OR ALL=(Parkinson's disease dementia)) AND ((ALL=(Argentina) OR ALL=(Bolivia) OR ALL=(Brazil) OR ALL=(Chile) OR ALL=(Colombia) OR ALL=(Costa Rica) OR ALL=(Cuba) OR ALL=(Ecuador) OR ALL=(El Salvador) OR ALL=(Guatemala) OR ALL=(Haiti) OR ALL=(Honduras) OR ALL=(Mexico) OR ALL=(Nicaragua) OR ALL=(Panama) OR ALL=(Paraguay) OR ALL=(Panama)) OR ALL=(Paraguay) OR ALL=(Peru) OR ALL=(Republica Dominicana) OR ALL=(Dominican Republic) OR ALL=(Uruguay) OR ALL=(Venezuela) OR ALL=(Puerto Rico))

Embase: ('parkinson dementia' OR 'diffuse lewy body disease'/exp OR 'diffuse lewy body disease') AND ('argentina'/exp OR 'argentina' OR 'bolivia'/exp OR 'bolivia' OR 'brazil'/exp OR 'brazil' OR 'chile'/exp OR 'chile' OR 'colombia'/exp OR 'colombia' OR 'costa rica'/exp OR 'costa rica' OR 'cuba'/exp OR 'cuba' OR 'ecuador'/exp OR 'ecuador' OR 'el salvador'/exp OR 'el salvador' OR 'guatemala'/exp OR 'guatemala' OR 'haiti'/exp OR 'haiti' OR 'honduras'/exp OR 'honduras' OR 'mexico'/exp OR 'mexico' OR 'nicaragua'/exp OR 'nicaragua' OR 'panama'/exp OR 'panama' OR 'paraguay'/exp OR 'paraguay' OR 'peru'/exp OR 'peru' OR 'dominican republic'/exp OR 'dominican republic' OR 'uruguay'/exp OR 'uruguay' OR 'venezuela'/exp OR 'venezuela' OR 'puerto rico'/exp OR 'puerto rico')

**Supplementary material 2.** Lewy body disease as a subsample in the study

There were some studies (n=34) that included patients with DLB or PDD in a broader and more diverse sample (46-75, 77-79, 81), addressing various aspects of the condition, including clinical manifestations, neuropsychological profiles, diagnostic tools and accuracy, non-pharmacological treatment and follow-up, biomarkers, supporting clinical tests, prevalence estimation and economic analysis, risk factors and predictors, as well as neuropathology and mortality (table 2). Patients were compared to AD in 14 studies (46 – 49, 51, 53, 55, 57, 61, 68, 69, 72, 77, 78), to FTD in 8 studies (48, 55, 72, 77, 56, 60, 69, 78), to VaD in 12 studies (46, 47, 49, 51, 53, 55, 57, 61, 68, 69, 72, 77), to mixed AD+VaD in 7 studies (46, 49, 53, 55, 57, 61, 77) and to PD in 18 studies (50, 52, 53, 58, 59, 60, 62-67, 71, 73-75, 79, 81). Moreover, other included copathologies were TDP-43 in neuropathological studies (47), or other disease in the parkinsonism spectrum such as corticobasal degeneration, multiple system atrophy, or progressive supranuclear palsy (50, 52, 75).

*Clinical manifestations*

Regarding non-motor symptoms, one study (50) reported that 74% of patients with DLB in the sample had probable rapid eye movement (REM) sleep behavior disorder. Also, PDD patients reported more cardiovascular symptoms than patients with PD alone (62). For motor symptoms, a study searched for facial tremors in patients with and without parkinsonism, and among the sample only found one patient with this symptom and DLB. Moreover, he did not respond to the levodopa challenge (59). As NPS is a frequent topic, a study analyzed the presence of depression in patients with PDD compared to patients with PD and showed that this symptomatology was directly associated with caregivers’ burden in patients with dementia (58). Finally, clinical and functional correlates of a large sample of patients with parkinsonism in Brazil were studied, and the ones with dementia had the worst cognitive and functional impairment as expected (70).

*Neuropsychological profile, diagnostic tools, and biomarkers*

One study (48) described some aspects of the neuropsychological evaluation of patients with LBD and found that they had unspecific differences in the Semantic Verbal Fluency (SVF) performance. Another diagnostic tool was electroencephalogram, as abnormalities were associated with mild cognitive impairment or dementia in patients with PD (63). Also, SPECT (single photon emission computed tomography) was used for the differential diagnosis of synucleinopathies (65).

Related to diagnostic, two studies (64, 66) assessed the performance of clinical tests on patients with DLB. We found evidence for the interlocking finger test (64), as well as the mentation, behavior, and mood section of The Unified Parkinson's Disease Rating Scale (66). A study done in Brazil (77) described that in general dementia patients’ diagnosis change, and 2 patients with PDD changed their diagnosis at follow-up.

One article evaluated biomarkers in CSF and found elevated CSF phospho-tau levels in one DLB patient; however, these levels were significantly higher in patients with Alzheimer’s disease compared to other dementias (69). Also, an expression of a-synuclein inclusion in skin cells included patients with DLB (81). Also, based on histological diagnosis, LBD was associated with cognitive impairment (47, 49).

*Non-pharmacological treatment and follow-up*

Regarding factors associated with physiotherapy in a sample of patients with PD, the higher educational level, the higher use of a physiotherapy service (71). In addition, telemedicine programs could reduce the financial burden for dementia patients of traveling to attend face-to-face consultations (53).

*Prevalence estimation and economic analysis*

Eleven studies had data on prevalence (51,52,54-56,60,61,73,74,81,83), with various specific samples as denominators (73, 74). Some examples are a university hospital (54), movement disorders clinics (56, 81), neurologic units of a tertiary hospital (60, 83) and specifically the Japanese-Brazilian population (51).

Also, some studies were population based, and could compare LBD patients with other parkinsonism such as progressive supranuclear palsy, or with other dementia etiologies such as AD. In Medellín, Colombia (52), in a sample of 302 patients with parkinsonism, 16 (5.4%) had DLB. In the Pieta study (54), it was found that DLB had a prevalence of 1% and for PDD it was 29.2%. In Sao Paulo, among 1563 patients, with 107 having dementia, only one case of DLB and one case of PDD were identified (57). And in Havana, Cuba, from a sample of 1499 patients with dementia, LBD prevalence was 0.3% (61).

A paper that evaluated direct and indirect costs of dementia in Brazil included one patient with DLB. In general, US$16,548.24 was the projected annual cost per patient, and factors that influenced this such as functional assessment and educational level of the caregiver were considered (72).

*Risk factors and predictors*

Taragano et al. (78), describe that neuropsychiatric symptoms may predict the conversion to dementia, with patients in the sample who had mild behavioral impairment progressing to DLB. Also, depression influences the relationship between PD and cognitive decline (67). Further, late-life depression and depressive symptoms were associated with brain infarcts and Lewy body disease (68).

*Mortality and neuropathology*

First, pneumonia as a cause of death was more prevalent in patients with DLB (46). Also, PDD itself is associated with mortality (79), but among patients with parkinsonism, the causes of death are similar (75).

Based on a pathology diagnosis, LBD was associated with cognitive impairment, and was compared to other neuropathological diagnosis such as AD, VaD or mixed AD+VaD (46, 47). Also, late life depression and depressive symptoms were associated with brain infarcts and Lewy body disease (68).

**Supplementary material 3 (Table). Full data on articles that were oriented to the study of patients with LBD.**

| **Reference** | **First author** | **Year** | **Country** | **Language** | **Study design** | **Number of patients with LBD** | **LBD diagnostic criteria** | **Age, mean (SD)** | **LBD Female % and n** | **MMSE or MOCA, mean (SD or range)** | **Main finding** |
| --- | --- | --- | --- | --- | --- | --- | --- | --- | --- | --- | --- |
| (14) | de Oliveira | 2020 | Brazil | English | Cross-sectional | DLB: 37 - PDD: 14 | MDS criteria and Fourth consensus of DLB consortium | 77.76 (7.8) | n=20, 39.2% (the whole sample) | NA | Higher burden of non-motor symptoms in DLB and PDD patients had anxiety and dysphoria when less burden of motor symptoms |
| (15) | Camargo | 2018 | Brazil | English | Cross-sectional | PDD: 34 | MDS criteria | NA | NA | NA | PDD patients with olfactory loss had increasing attention impairment and increasing executive-function impairment |
| (16) | Garcia Basalo | 2017 | Argentina | English | Cross-sectional | DLB: 75 | Third consensus of DLB consortium | 74.7 ± 7 | 52%, n=39 | MMSE: 25.2 ± 3 MOCA: 20.4 ± 5 | ASI has a sensitivity of 90.7% and a specificity of 93.6% for LBD. It can be performed by non-medical staff. |
| (17) | Rocha | 2014 | Brazil | English | Cross-sectional | PDD: 37 | MDS criteria | 67.48 ± NA | 59.3%, n=16 | MMSE: 20.7 ± 3.9 | Addenbrooke's Cognitive Examination-Revised had good performance por PDD evaluation in a sample of patients with differents educational level |
| (80) | Golimstok | 2011 | Argentina | English | Case control | DLB: 109 | First consensus of DLB consortium | 75.1 ± 7.4 | 67.4%, n=73 | MMSE: 21.7 ± 4.6 | Patients with DLB had a higher chance of having symptoms of attention-deficit and hyperactivity disorde, with an OR of 5.1 (IC95% 2.7-9.6), compared to the control group |
| (18) | Sobreira | 2015 | Brazil | English | Cross-sectional | PDD: 17 | MDS criteria | 72.5 (53-81) | 76.92% (n=10, from total PPD n=13) | MOCA: 17 (7-24)± | MOCA and ACE-R scales showed to be useful to screen for dementia but not for mild cognitive impairment in patients with PD. |
| (82) | Custodio | 2008 | Peru | Spanish | Open essay clinical trial | PDD: 21 - DLB: 12 | DSM IV criteria, First consensus of DLB consortium | Rivastigmine: 74,2 (7,7)  Donepezil: 74,4 (6,9)  Galantamine: 72,8 (8,2) | 33%, n=11 | Rivastigmine: 16,6 (2,9) Donepezil: 16,2 (3,1)  Galantamine: 16,4 (3,1) | Cholinesterase inhibitors may be of benefit for DLB and PDD, especifically in global function and daily living activities. |
| (19) | Pérez | 2000 | Cuba | Spanish | Cross-sectional | PDD: 19 | DSM IV criteria | 71.1 ± 5.36 | NA | NA | Intensity of parkinson disease and the educational level of the patient were associated with dementia. |
| (20) | Machado | 2020 | Brazil | English | Cross-sectional | PDD: 20 - DLB: 22 | MDS criteria | 75.84 ± 9.1 | 59.4%, n=19 | MMSE 17.72 ± 5.7 | Linguistic impairments in LBD may stem from various cognitive and language-related factors, also, neuropsychiatric symptoms may have distinct relationships with visual organization compared to linguistic features in LBD. |
| (76) | De Oliveira | 2023 | Brazil | English | Cross-sectional | DLB: 27 | Fourth consensus of DLB consortium | 78.98 ± 9.0 | 66.7%, n=18 | MMSE: 15.56 ± 5.0 | Cerebrospinal fluid phospho-tau Thr181 in DLB was similar to AD, but not Aβ42. In associations with test scores, biomarker ratios were superior to isolated biomarkers, while worse 22)functionality was associated with axonal degeneration only in AD. |
| (22) | Clavijo-Moran | 2022 | Colombia | English | Cross-sectional | NA | MOCA score <18 | NA | NA | MoCA≤17 | PD-CRS has a significant correlation and agreement with the MoCA, and acceptable psychometric properties |
| (23) | Sobreira | 2019 | Brazil | English | Cross-sectional | PDD: 11 | MDS criteria | 69.0± 9.6 | 9.7%, n=3 | NA | PDD patients had a lower sleep efficiency, lower total sleep time, and lower number of sleep state changes in comparison to the normal cognition group, also the wake time after sleep onset and the number of state changes during sleep have a significant association with global cognitive performance. |
| (24) | Sousa | 2023 | Brazil | English | Cross-sectional | PDD: 22 | MDS criteria | 66.50 ± 9.24 | 50%, n=11 | NA | ACE-III is a useful battery for assessing the cognitive domains and to differentiate individuals with MCI-PD and D-PD from healthy controls. |
| (83) | Camargo | 2019 | Brazil | English | Non randomized clinical trial | PDD: 12 | MDS criteria | 72.08 ± 13.53 | 41.67%, n=5 | NA | Reality orientation therapy can potentially be used to complement drug therapy in patients with PDD, it showed improvement in cognitive function with a weekly individual session for 6 months. |
| (25) | Camargo | 2016 | Brazil | English | Cross-sectional | PDD: 39 | MDS criteria | 70.7±10.93 | 35.9%, n=14 | NA | Apathy is a disorder associated with PDD, 97.4% had high scores on the AES, however the instrument had several limitations. |
| (26) | Reyes | 2009 | Argentina | English | Cross-sectional | PDD: 13 | MDS criteria | 71.9 ± 10.5 | 31%, n=4 | MMSE: 25.2 ± 2.7 | Addenbrooke’s Cognitive Examination appears to be a valid tool for dementia evaluation in PD, with a cut-off point which should probably be set at 83 points, displaying good correlation with both the scale specifically designed for cognitive deficits in PD namely SCOPA-COG, as well as with less specific tests such as MMSE. |
| (27) | Almeida | 2019 | Brazil | English | Cross-sectional | PDD: 25 | MDS criteria | 65.48 ± 8.67 | 56%, n=14 | MOCA; 15.28 ± 3.6 - MMSE: 20.56 ± 4.75 | The cutoff score to distinguish normal cognition from PDD was 22.5 and MCI from PDD was 17.5, both the sensitivity and specificity were higher than 70%. |
| (28) | Camargo | 2018 | Brazil | English | Cross-sectional | PDD: 33 | MDS criteria | 62.3 ± 11.6 | 39.4%, n=13 | NA | The CERAD may have the necessary efficacy and accuracy for the evaluation of cognition in PD patients when compared to Clinical Dementia Rating Scale |
| (29) | Souza | 2016 | Brazil | English | Cross-sectional | PDD: 40 | MDS criteria | 69 (46-87) | NA | MMSE: 21 (9-29) | Cognitive impairment in PD occurs progressively and heterogeneously in most patients, the initial phenotype cannot be used to stablish prognosis. The patients with dementia had lower education and lower MMSE scores |
| (30) | Tumas | 2016 | Brazil | English | Cross-sectional | PDD: 29 | MDS criteria + MOCA score <21 | NA | NA | NA | Some MoCA subtests are too difficult to be completed by patients with lower education thereby contributing to the test's poor diagnostic accuracy, also patients with dementia had longer disease duration and lower education. |
| (31) | Schelp | 2016 | Brazil | English | Cross-sectional | PDD: 46 | MDRS | NA | NA | NA | There is a significant relationship between age and dementia characterized by impaired episodic memory. |
| (32) | Campos | 2015 | Brazil | English | Cross-sectional | PDD: 28 | SCOPA-COG Score ≤17 | NA | NA | NA | Level of education and disease severity are predictors of dementia in PD. |
| (33) | Custodio | 2013 | Peru | Spanish | Cross-sectional | PDD: 23 | DSM IV criteria | 71.4 ± 7.1 | 34.78%, n=8 | NA | Patients with Parkinson's disease exhibit, depending on the disease progression, a progressive compromise of cognitive areas. |
| (34) | Schelp | 2012 | Brazil | English | Cross-sectional | PDD: 19 | MDRS | NA | NA | NA | There was no evidence to correlate the presence of metabolic syndrome with the risk of dementia that was associated with PD. Dementia in PD is age dependent and not related to disease duration. |
| (35) | Tedrus | 2009 | Brazil | English | Cross-sectional | PDD: 7 | CDR | 70.6 ± 9.1 | NA | MMSE: 12.9 ± 3.3 | Dementia and mild cognitive impairment frequently occur in PD patients and should be investigated in a routine way. |
| (36) | Gibson | 2023 | Brazil | English | Cross-sectional | LBD: 60 - AD+LBD: 28 | Braak Parkinson's disease stage ≥ 3 and CDR | Age at dead: 78.8 ± 7.67 | 40.4%, n=23 | NA | Cases with dual AD+LBD pathology had the highest risk of hallucinations, agitation, apathy, and total symptoms but a multiplicative interaction between these pathologies was not significant. |
| (37) | Oliveira | 2015 | Brazil | English | Cross-sectional | PDD: 33 | MDS criteria | NA | NA | NA | To improve the accuracy of the MDS checklist, it would be necessary to adjust the way we use and interpret the cut-off scores of the MMSE and of the subtests, without the need to eliminate their use. |
| (38) | Calil | 2021 | Brazil | English | Cross-sectional | DLB: 20 | Fourth consensus of DLB consortium | 77.1 ± 5.53 | n=9 | NA | DLB patients had a worse awareness for memory deficits. |
| (21) | de Oliveira | 2021 | Brazil | English | Cross-sectional | DLB: 27 | Fourth consensus of DLB consortium | 78.48 ± 9 | 66.7%, n=18 | MMSE: 15.56 ± 5 | Larger behavioral burden in DLB, especifically in hallucinations and apathy |
| (39) | Ferreira Camargo | 2017 | Brazil | English | Cross-sectional | PDD: 40 | MDS criteria | 70.075 ± 10.80 | 35.0%, n=14 | NA | Depression was associated with less advanced PDD and more intense motor features, while apathy was associated with more advanced cognitive impairment. |
| (40) | Josviak | 2017 | Brazil | English | Cross-sectional | DLB: 18 | Third consensus of DLB consortium | 74.5±8.52 | 50%, n=9 |  | Butyrylcholinesterase has a lowe plasma activity in patients with DLB |
| (41) | Oliveira | 2015 | Brazil | English | Cross-sectional | DLB: 25 - PDD: 14 | Third consensus of DLB consortium and MDS criteria | DLB: 70.86 ± 9.09 and PDD: 73.14 ± 9.36 | DLB: 52% (n=13); PDD: 64.3% (n=9) | MMSE: DLB: 18.16 ± 5.72 and PDD: 16.79 ± 7.37 | LBD patients were more oriented, and related to neuropsyciatric symptoms, hallucinations, apathy, dysphoria, anxiety, and aberrant motor behavior were the most significant to diffirenciate with AD, the latter having lower CDR scores |
| 423) | Tabernero | 2017 | Argentina | Spanish | Cross-sectional | PDD: 34 | MDS criteria | 70.18 ± 8.7 | 38.3%, n=13 | NA | The theory of mind test is more affected in patients with PDD, and in general all the used social cognition tools were impaired |
| (43) | Fonseca | 2013 | Brazil | English | Cross-sectional | PDD: 12 | MDS criteria | 70.3 ± 11.9 | 66%, n=8 | MMSE: 14.5 ± 5.6 | Delta and Theta powers, beta frontal-occipital inter-hemispheric coherence, alpha and beta frontal inter-hemispheric coherence was highest in PDD patients |
| (44) | Espínola Nadurille | 2007 | Mexico | Spanish | Cross-sectional | DLB: 1 | DSM IV criteria | NA | NA | NA | In underdeveloped or developing countries, vascular pathology could modify the clinical presentation of neurodegenerative processes, as occurs with Lewy body dementia |
| (45) | Lourenco | 2021 | Brazil | English | Cross-sectional | DLB: 9 | Not specified | 73.7 ± 6.7 | n=7 | MMSE: 21.7 ± 3.2 | Homovalinic acid and vascular endotelial growth factor were reduced in DLB patients |

**Supplementary material 4 (Table). Full data on articles that had patients with LBD in the sample.**

| **Reference** | **First author** | **Year** | **Country** | **Language** | **Study design** | **Number of patients with LBD** | **LBD diagnostic criteria** | **LBD Age, mean (SD)** | **LBD Female % and n** | **LBD MMSE or MOCA, mean (SD)** | **Main finding** |
| --- | --- | --- | --- | --- | --- | --- | --- | --- | --- | --- | --- |
| (46) | Astolfi Neves | 2022 | Brazil | English | Cross-sectional | DLB: 53 | Braak Parkinson's disease stage ≥ 3 and CDR | 78.2 ± 7.8 | 43.4%, n=23 | NA | Pneumonia as cause of death was more prevalent in patients with DLB = 2.25 (1.03–4.93) |
| (47) | Suemoto | 2019 | Brazil | English | Cross-sectional | LBD: n=25 for <80 years and n=37 for ≥80 years | Braak Parkinson's disease stage > 3 and CDR | NA | NA | NA | LBD associated with cognitive impairement in old population, the last having also a larger odd of having multiple neuropathological diagnosis |
| (48) | Wajman | 2019 | Brazil | English | Cross-sectional | DLB: 22 | Fourth consensus of DLB consortium | 72.05 ± 5.87 | 45.5%, n=10 | MMSE: 23.41 ± 2.86 | LBD patients had unspecific differences in the Semantic Verbal Fluency (SVF) performance |
| (49) | Suemoto | 2017 | Brazil | English | Cross-sectional | LBD: 87 | Braak Parkinson's disease stage ≥ 3 // Third consensus of DLB consortium and International Parkinson and Movement Disorder Society (MDS) criteria | NA | NA | NA | Neuropathological presence of LBD was associates with cognitive impairement; and compared to this method of diagnosis, clinical diagnosis high specifity for LBD |
| (50) | Munhoz | 2014 | Brazil | English | Cross-sectional | DLB: 50 | Third consensus of DLB consortium | 74 ± 6.2 | 44%, n=22 | NA | 74% of the patients with DLB had probable REM sleep behavior disorder |
| (51) | Yamada | 2002 | Brazil | English | Cross-sectional | PDD: 1 | Third consensus of DLB consortium and DSM III-R criteria | NA | NA | NA | From 157 examined persons, only one had PPD, no cases of DLB were found |
| (52) | Pineda | 2000 | Colombia | English | Cross-sectional | LBD: 16 | First consensus of DLB consortium | NA | NA | NA | In a sample of 302 patients with parkinsonism, 16 (5.4%) had DLB |
| (53) | Pessoa | 2022 | Peru | English | Cross-sectional | DLB: 3 - PDD: 2 | DSM V Criteria | NA | NA | NA | The program showed a high rate of attendance and satisfaction and also reduced the finantial burden of traveling to the dementia patients |
| (54) | Vale | 2018 | Brazil | English | Cross-sectional | PDD: 5 - DLB: 1 | DSM IV Criteria | NA | NA | NA | Parkinsonism was common in this oldest-old, but DLB had a prevalence of 1% and for PDD 29.2% |
| (77) | de Moraes | 2017 | Brazil | English | Cohort | DLB: 27 - PDD: 18 | Not specified | NA | NA | NA | 113 (26.6%) patients have their diagnosis changed, mostly adding a vascular component or depression, 2 patients with PDD changed their diagnostic in the followup |
| (81) | Rodríguez-Leyva | 2014 | Mexico | English | Case control | DLB: 12 | Third consensus of DLB consortium | NA | NA | NA | PD patients had a relative high expresion a-synuclein compared to other groups |
| (55) | Vieira | 2013 | Brazil | English | Cross-sectional | DLB: 8 | First consensus of DLB consortium | NA | NA | NA | VD, AD and LBD are the most common subtypes observed at both groups, DLB had a higher frequency than AD. |
| (56) | Rodríguez-Violante | 2012 | Mexico | Spanish | Cross-sectional | DLB: 10 | Third consensus of DLB consortium | NA | NA | NA | DLB had a frequency of 27.2% among the atypical parkinsonism, and 1.5% among all parkinsonism |
| (57) | Bottino | 2008 | Brazil | English | Cross-sectional | DLB: 1 - PDD: 1 | DSM IV criteria | NA | NA | NA | Among 1563 patients, with 107 having dementia, only one case of DLB and one of PDD were found. |
| (58) | Stella | 2009 | Brazil | English | Cross-sectional | PDD: 13 | DSM IV criteria and MDS criteria | 70.7 ± 7.0 | NA | NA | Patients with dementia and depression have a highest NPI. Also symptomatology was directly assciated with caregivers burden. |
| (59) | Rossi | 2016 | Argentina | English | Cross-sectional | DLB: 1 | Not specified | 69 | 0% | NA | There was one patient with facial tremor and DLB, bur he did nor respond to the levodopa challenge |
| (60) | Neto | 2017 | Brazil | English | Cross-sectional | DLB: 1 | Not specified | NA | NA | NA | Unlike other studies were neurodegenerative diseases are the most common nonprion etiologies, in this study only 5 patients were diagnosed with degenerative diseases (1 probable Lewy body disease) |
| (61) | Llibre | 2009 | Cuba | English | Cross-sectional | DLB: 5 - PDD: 33 | DSM IV criteria and Operational criteria for SDLT | NA | NA | NA | From a sample of 1499 patients with dementia, LBD prevalence was 0.3% |
| (62) | Idiaquez | 2007 | Chile | English | Cross-sectional | PDD: 11 | DSM IV criteria | NA | NA | NA | The presence of ortostatic hypotension or postprandial hypotension did not correlate with the severity of cognitive impairment in our PD cases. However, PD patients with dementia reported more cardiovascular symptoms than PD patients without dementia. |
| (63) | Fonseca | 2009 | Brazil | English | Cross-sectional | PDD: 7 | DSM IV criteria | 70.6 ± 9.1 | NA | NA | Abnormalities on the qEEG were essentially associated with the occurrence of mild cognitive impairment or dementia in patients with PD. |
| (64) | Souza | 2016 | Brazil | English | Cross-sectional | PDD: 41 | MDS criteria | 68.8 ± 9.7 | NA | MMSE: 21.4 (3.8) | ILFT scores were significantly lower in patients with PD and dementia and significantly correlated with cognitive and functional tests, but not with depressive symptoms, Hoehn and Yahr scores, or Schwab and England scores. |
| (65) | Fabiani | 2022 | Brazil | English | Cross-sectional | DLB: 16 | Fourth consensus of DLB consortium | 72.8 ± 7.6 | 50%, n=8 | NA | A direct relationship was found between motor scores severity (MDS-UPDRS-III) and SPECT-TRODAT-reduced binding in general, in the group of patients with synucleinopathies, especially in patients with Parkinson's disease. |
| (78) | Taragano | 2018 | Argentina | English | Cohort | DLB: 28 | First consensus of DLB consortium | NA | NA | NA | Conversion to dementia is significantly higher in patients with neuropsychiatric symptoms. Mild cognitive impairment patients mostly converted to Alzheimer's dementia, while Mild behavioral impairment converted to frontotemporal dementia and DLB. |
| (66) | Starkstein | 2007 | Argentina | English | Cross-sectional | PDD: 62 | DSM IV criteria | NA | NA | NA | An ≥2 score on the intellectual impairment item of the UPDRS and a <23 score on the MMSE had a good sensitivity (up to 85%) to detect dementia. |
| (67) | Chagas | 2015 | Brazil | English | Cross-sectional | PDD: 24 | DSM IV criteria and CSI-D | NA | NA | NA | The association between depression and PD increases with the severity of the cognitive impairment. |
| (68) | Nunes | 2022 | Brazil | English | Cross-sectional | LBD: 68 | Braak Parkinson's disease stage ≥ 3 and CDR | NA | NA | NA | Late life depression and depressive symptoms were associated with brain infarcts and Lewy body disease |
| (69) | Hartmann | 2014 | Brazil | English | Cross-sectional | DLB: 2 | First consensus of DLB consortium | NA | NA | NA | Even though the study found one DLB patient with CSF phosphotau levels elevated, these levels are significantly higher in patients with Alzheimer disease compared to other dementias. |
| (79) | Fernandes | 2015 | Brazil | English | Cohort | PDD: 51 | Minimental State Exam ≤23 | NA | NA | NA | Dementia and cognitive impairment are well-established risk factors for mortality in PD patients and there is not enough data to confirm that these features increase with age. |
| (70) | Vale | 2023 | Brazil | English | Cross-sectional | PDD: 37 | BCSB, FAQ scores and DSM IV criteria. | NA | NA | NA | Patients with parkinsonism had a significantly more frequent diagnosis of dementia and their cognitive performance and functionality were also significantly more impaired compared to individuals without parkinsonism. |
| (74) | Munhoz | 2010 | Brazil | English | Cross-sectional | DLB: 42 | Third consensus of DLB consortium and DSM IV criteria | 75.3 ± 5.2 | 52.4%, n=22 | NA | Among parkinsonisms, Lewy body dementia was the least common differential diagnosis, with a prevalence of 2.7%. |
| (73) | Balestrassi | 2021 | Brazil | English | Cross-sectional | LBD: 4 | Not specified | NA | NA | NA | Parkinson’s disease, essential tremor and dystonia were the most frequent diagnoses. Among all patients, there were just 4 patients with DLB diagnosis. |
| (71) | Rodrígues-de-Paula | 2018 | Brazil | English | Cross-sectional | PDD: 230 | MOCA score < 21 | NA | NA | NA | In Brazil a higher educational level increased the use of a physiotherapy service. |
| (72) | Ferretti | 2018 | Brazil | English | Cross-sectional | DLB: 1 | Not specified | NA | NA | NA | Annual costs of dementia in Brazil are projected to be 16 584.24 US$ |
| (75) | Moscovich | 2017 | Brazil | English | Cross-sectional | DLB: 10 and PDD: 42 | Third consensus of DLB consortium and MDS criteria | NA | NA | NA | Patients with parkinsonism have a similar cause of death |
